# Insights into AI and facial ageing

**DOI:** 10.1111/jdv.20401

**Published:** 2024-11-25

**Authors:** Tamara W. Griffiths

**Affiliations:** ^1^ University of Manchester and Northern Care Alliance NHS Foundation Trust Manchester UK

Above all else, the article ‘Deep learning predicted perceived age is a reliable approach for analysis of facial ageing: a proof of principle study’^1^ underscores two key questions emerging in dermatology. The more pressing is: how can artificial intelligence (AI) be applied in an accurate and safe manner to reduce the burden of work on an already overstretched dermatology workforce? The second, a longer‐term interrogation on health span: what are the implications of using a patient's facial image and perceived age as a marker for intrinsic ageing, to prognosticate morbidity and mortality?

With regard to the first question, one does not have to be a mathematician, statistician or computer scientist to understand AI has tremendous potential for application in our visually‐orientated specialty. However, the input to algorithms and deep learning image technology hinge on these key disciplines, combined with clinician input to drive accurate databases from which the systems learn and function. High‐speed processing results in rapid escalation of learning but likewise, small undetected miscues can be magnified exponentially, with potentially disastrous impact.

This pilot study implements two computational paradigms which score perceived age from facial photographs. One trained on perceived age data scored by humans, the other on repurposed features of face recognition technology. The authors convincingly validate estimations from both of these models using morbidity and genetic markers such as osteoporosis, cataracts and the *MCR1* gene, as intermediaries correlated to human‐scored facial images.

This is encouraging, but fundamental issues which apply to a number of AI models remain relevant. Training on repurposed or recursively generated data can result in the collapse or corruption of AI models,^2^ therefore, continued learning from datapoints driven by clinician input is critical. Extrapolation of potential application to smartphone or selfie images at this stage is premature and its mere suggestion incautious. As clinicians and scientists we have some responsibility in managing the unchecked drift towards over‐eager and unproven implementation of AI in dermatology, or at least must refrain from contributing to it.

Finally, as with much of AI in dermatology, the nuances of racial and ethnic variations are unaddressed. One assumes volunteers from a population‐based study in the Netherlands to be of homogenous northern European ancestral origin, otherwise, the outcome data may be irrevocably skewed rendering them ineffectual. However, by omitting any reference to this accentuates another real concern with AI expansion, apposite to both clinical service and research: the under‐representation of patients with skin of colour.^3^

The second question is significant with strategic implications. It holds substantial weight in a world with an ageing population and real need for tools to support preventative medicine and health span. The concept that external appearance related to skin and facial ageing can accurately point to or predict disease in other organ systems^4^ is exciting and a potential game‐changer for dermatology.

The natural follow‐on questions are: what impact does improvement of an individual's perceived age have on systemic health? Intuitively cosmetic procedures would invalidate the true perceived age score, but can manipulation and repair of sun damage and senescence at the cellular level in the body's largest organ system—the skin—impact internal cytokine cascades, inflammaging and subsequently systemic diseases?

It may seem a fanciful and far‐fetched concept, but over the past decades, dogma that psoriasis is merely a skin disease has been challenged, with recognized links to metabolic syndrome and other systemic morbidities. There is even early evidence to suggest the aggressive management of psoriasis may improve cardiovascular health.^5^ The corollary hypothesis that reducing cutaneous inflammation caused by photodamage or skin senescence equates to systemic reparative effects is less clear, but it is a question worth investigating. Any tools, AI or otherwise, which accurately and reliably support or facilitate this research should be encouraged.

## CONFLICT OF INTEREST STATEMENT

Conflicts of interest over the past 5 years: Research Grant: Walgreen Boots Alliance. Research Grant: Allergan, Evolus. Speaker fees: L'Oreal, Almirall. Trustee: British Association of Dermatologists. Trustee: Cosmetic Practice Standards Authority. Shareholder: The Skin Diary (formerly CGskin).

## Data Availability

Data sharing is not applicable to this article as no new data were created or analysed in this study.
